# Diagnostic Performance of Rapid Antigen Tests to Detect Equine Rotavirus A

**DOI:** 10.3390/v17030413

**Published:** 2025-03-14

**Authors:** Ann Cullinane, Maura Nelly, Laura Dayot, Gabija Lukaseviciute, Marie Garvey, Jennifer Healy, Robert Gallagher

**Affiliations:** Virology Unit, The Irish Equine Centre, Johnstown, Naas, Co., W91 RH93 Kildare, Ireland; mnelly@irishequinecentre.ie (M.N.); ldayot@irishequinecentre.ie (L.D.); glukaseviciute@irishequinecentre.ie (G.L.); mgarvey@irishequinecentre.ie (M.G.); jhealy@irishequinecentre.ie (J.H.); gallagher1292@gmail.com (R.G.)

**Keywords:** equine, rotavirus, diagnosis, antigen, kit, real-time RT-PCR

## Abstract

This study describes a comparison of the detection of rotavirus in clinical samples from foals using two commercially available rapid antigen detection (RAD) kits, with the detection of rotavirus nucleic acid via a laboratory-based, in-house, real-time reverse transcription polymerase chain reaction (RT-PCR) assay. One hundred and forty freeze-thawed samples (70 that were RT-PCR-positive and 70 that were RT-PCR-negative on original tests) submitted to the diagnostic laboratory over a seven-year period were tested in addition to 123 fresh samples (15 RT-PCR-positive and 108 RT-PCR-negative) submitted over a four- month period in 2024. The analyst performing the RAD tests was blinded to the RT-PCR result as were the two individuals who read the results. Samples with discordant results were re-tested in duplicate using RT-PCR and the two RAD kits. Both kits demonstrated a high level of concordance with the RT-PCR (>95%). However, testing of serial dilutions of RT-PCR positive faeces samples indicated that the RADs failed to detect the virus at the higher dilutions. In conclusion, the RADs evaluated are potentially useful for screening individual foals and for the determination of the urgency of the appropriate treatment and isolation. Negative samples from suspect cases and weak positives should always be submitted to a specialist laboratory for real-time RT-PCR testing.

## 1. Introduction

Rotavirus is a major cause of diarrhoea in young children [1] and many mammalian [2,3] and avian [4] species globally. The rotavirus genome consists of 11 segments of double-stranded RNA that encode six structural and six non-structural proteins. The outer glycoprotein VP7 which contains major neutralising epitopes, and the protease-cleaved VP4 are used to classify rotaviruses into genotypes, for example, G3P[12] and G14P[12] that predominate in horses globally [5,6]. The virus is transmitted predominantly through the faecal–oral route and is potentiated by the low infectious dose and high concentration in the faeces [7]. Infection results in the destruction of the middle and tip of intestinal villi and osmotic diarrhoea due to malabsorption [8]. Activation of the enteric nervous system also plays a role in the pathogenesis [9].

Equine rotavirus A is ubiquitous and estimated to be responsible for more than 25% of cases of foal diarrhoea [2]. A previous study in Ireland of 438 diagnostic samples from foals with enteric disease found that 23% tested positive for rotavirus [10]. The seasonality of the disease correlates with the time of year that stud farms are busiest with the foaling and breeding of mares. Rotaviruses are highly resistant and can survive for several months in a contaminated environment [11,12]. Some farms have recurrent problems with rotavirus infection. This can be due to poor hygiene but may also occur on premises with good biosecurity practices, but dense stocking rates. High-density stocking facilitates environmental contamination, foal-to-foal contact, and the transfer of the virus by humans, flies and fomites [13,14].

The foals of visiting mares are often more susceptible than the foals of resident mares. Clinical signs include reluctance to suck, lethargy, pyrexia, diarrhoea, dehydration and recumbency [15]. The incubation period is short, and diarrhoea may occur within 24 h of exposure to the virus [16]. The disease is associated with high morbidity but low mortality rates. The consequences of rotavirus infection appear to be strongly associated with age [17,18]. Older foals may have subclinical infection or exhibit mild clinical signs [19]. Equine rotavirus is not associated with disease in adult horses [20]. Foals less than five months of age are most susceptible and those under two weeks of age can develop life-threatening dehydration within hours. The management of rotavirus infection focuses on early diagnosis and the prevention of dehydration [19]. Since the COVID-19 pandemic, there is greater awareness of the potential role of rapid antigen testing in the detection of viral disease [21,22]. This study describes a comparison of the detection of rotavirus antigen in clinical samples by immunochromatography using commercially available kits, with the detection of rotavirus nucleic acid via an in-house real-time reverse transcription polymerase chain reaction (RT-PCR)-based assay.

## 2. Materials and Methods

### 2.1. Experimental Design

The detection limits of the two rapid antigen detection (RAD) kits and the RT-PCR in four diagnostic samples were estimated using serial ten-fold dilutions in a kit buffer or nuclease-free water (NFW) as appropriate. Each dilution was tested in triplicate. Seventy faeces samples that had previously tested positive for rotavirus A by real-time RT-PCR and 70 faeces samples that had tested negative by real-time RT-PCR were randomised in Microsoft Excel 2013 and tested with two commercial rapid antigen detection (RAD) kits. The samples were submitted to the diagnostic laboratory between 2018 and 2024 and were stored at −70 °C prior to inclusion in the study. The analyst performing the RAD tests was blinded to the RT-PCR results as were the two individuals who read the results. Samples with discordant results were re-tested in duplicate via RT-PCR and the two RAD kits unless there was insufficient sample. In the case of samples that were retested, the majority result was accepted. The performance of the RADs was also evaluated on 123 fresh samples submitted to the diagnostic laboratory over a four-month period in 2024.

### 2.2. Real-Time RT-PCR

Faeces samples were mixed with a disposable loop (Ref 302774 Medline Scientific, Chalgrove, UK) and sampled in four different parts of the faecal matter and added to 800 µL of NFW. After mixing to achieve homogeneity, the sample was allowed to stand for 5 min with occasional inversion and then was centrifuged at 166× *g* for 5 min. A 1:10 dilution of the supernatant was made in NFW. RNA was extracted from 200 µL of the supernatant (neat) and a 1:10 dilution of supernatant using the Kingfisher Flex Magnetic Particle Processor instrument (Thermo Scientific/Life Technologies, Singapore) with the MagMax Core Nucleic Acid Purification kit (Cat No: A32702, Applied Biosystems by Thermo Fisher Scientific, Austin, TX, USA) using the complex workflow as per the kit manufacturer’s guidelines. An exogenous RNA control (Ref: Z-INT-RNA-VIC Primer Design Ltd., Chandler’s Ford, UK) was included in the extraction process.

An in-house real-time RT-PCR targeting the non-structural protein NSP5 (equine rotavirus A-NSP5 16F- CAGTGATGTCTCTCAGTATTG, equine rotavirus A-NSP5-147R GTGAAATGTATTGTTCACTCCTAC, and * equine rotavirus A-NSP5 Probe-6FAM-CAACGTCGACTCTTTCTGG-MGB) was used to confirm equine rotavirus A as the etiologic agent using the TaqMan 7500 Real-Time PCR with the AgPath-ID™ One-Step RT-PCR kit (Applied Biosystems by Thermofisher, Austin, TX, USA). The reaction component (25 µL) for amplification consisted of 12.5 µL of 2X Buffer, 2 µL of tRNA, 0.4 µM of each primer, 0.16 µM of probe, 0.9 µL of internal control primer/probe mix (Ref: Z-INT-RNA-VIC Primer Design Ltd., Chandler’s Ford, UK), 1 µL of 25X enzyme, nuclease-free water and 5 µL of template RNA. The cycling conditions were as follows: reverse transcription at 45 °C (10 min), initial denaturation at 95 °C (10 min), followed by 40 cycles of denaturation at 95 °C (15 s) and annealing at 60 °C (45 s, data collection).

### 2.3. Rapid Antigen Detection (RAD) Tests

Samples were tested by two commercial immunochromatographic kits, the CerTest Rotavirus and Adenovirus (Biotec S.L, Zaragoza, Spain) and the FASTest Rota strip (MEGACOR, Diagnostk GmbH, A-6912 Hörbranz, Austria), used for the qualitative detection of Rotavirus A antigens in human and animal faeces, respectively. The CerTest Rotavirus and Adenovirus can be used for the simultaneous detection of Rotavirus and Adenovirus, however only Rotavirus was of interest in this study. The test kits and faeces samples were at room temperature (15–30 °C) before the tests commenced. Samples were homogenised thoroughly with an applicator stick or loop before testing.

Using the CerTest Rotavirus and Adenovirus kit, the stick was introduced into four different parts of the stool sample to collect a representative sample to add to the diluent in the collection tube. For liquid samples, approximately 125 µL of sample was added to the diluent. After shaking to ensure good sample dispersion, four drops of the solution were added to the rotavirus test window. The test results were read after 10 minutes.

The required sample volume used with the FASTest Rota strip varies depending on the consistency of the sample. For compact, pulpy and fluid-watery faeces, one, two and three level spoons, respectively, were added into the buffer diluent. After rotating to homogenise the solution, the tube was left to stand for 5 min to allow the sedimentation of gross faecal particles. The dipstick was inserted vertically into the sample tube for at least one minute and removed from the sample buffer mixture as soon as it reached the control line. The dipstick was then incubated on a flat surface and the results were read after 5–8 (maximum 10) minutes.

For both test kits, a test band and control band appearing together indicated a positive result. The presence of only a control band indicated a negative result. The diagnostic sensitivity and specificity of the two RADs compared with RT-PCR were calculated as per the WOAH Terrestrial Manual (2023) [23]. The concordance rate was calculated as per Nemoto et al. 2010 [24].

## 3. Results

**Figure 1:**
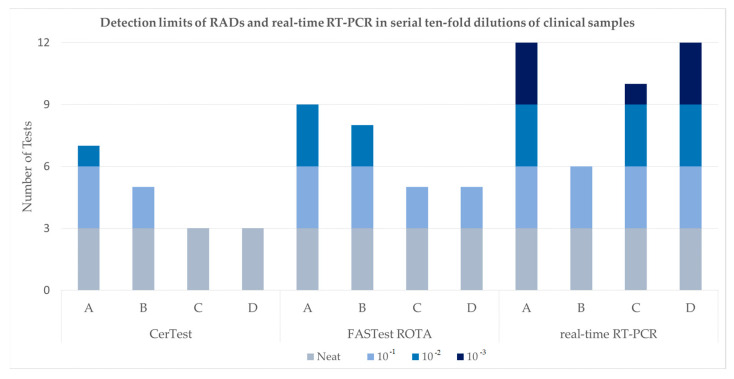
Graphical summary of results in Table 1 showing the detection limits of two RADs (CerTest and FASTest) and the in-house real-time RT-PCR in serial ten-fold dilutions of clinical samples A–D.

On testing serial dilutions of four faeces samples, an in-house real-time RT-PCR exhibited the highest sensitivity and the FASTest ROTA test was more sensitive than the CerTest (see Table 1 and Figure 1). Both RADs and RT-PCR detected the undiluted sample without fail. The CerTest failed to detect two samples at 10^−1^ which were detected in two of three tests with the FASTest ROTA and on all three tests by RT-PCR (Ct 33).

At a 10^−2^ dilution, the CerTest failed to detect any positives except for one of three tests on a single sample. The FASTest ROTA detected two samples and the RT-PCR detected three samples (Ct 35, 37 and 36). At 10^−3^, only the RT-PCR detected positive samples (Ct 38). Of the 60 tests performed, the CerTest was positive for 18, the FASTest ROTA was positive for 27 and the RT-PCR was positive for 40. Supplementary Figure S1a–h consist of photographs of RAD results for preliminary tests performed with samples A–D.

One hundred and forty convenience samples, i.e., samples submitted to the diagnostic laboratory over a seven-year period and stored at −70 °C, were selected for inclusion in the study. This study population consisted of 70 samples that originally tested negative and 70 that tested positive by RT-PCR. However, after freeze-thawing and repeating the RT-PCR for the 11 samples that had discrepant results, 3 of the 70 initially positive samples tested negative in duplicate (Supplementary Table S1). Thus, for the purposes of comparing the diagnostic sensitivity and specificity of the three tests, the status of the study samples was revised to 73 negative and 67 positive (Table 2 and Table 3). After this reclassification, eight samples had RAD results that differed from their RT-PCR results. Three RT-PCR-positive samples tested negative on both RADs and two tested negative on one RAD. Three RT-PCR-negative samples tested positive on one RAD. The discrepant test results for the eight samples were confirmed through repeat testing. Sample information and results are presented in Supplementary Table S1.

All of the seventy-three samples that tested negative via RT-PCR tested negative on the FASTest but three tested positive on the CerTest. Of the sixty-seven samples that tested positive via RT-PCR, three tested negative on the CerTest and five tested negative on the FASTest ROTA. These results are summarised in Table 2 and Table 3. The discrepant results were confirmed in duplicate for all three tests. The diagnostic sensitivity and specificity of the CerTest compared to RT-PCR was 95.7% and 96.1%, respectively, resulting in a concordance rate of 95.7%. The diagnostic sensitivity and specificity of the FASTest compared to RT-PCR was 93.1% and 100%, respectively, resulting in a concordance rate of 96.4%.

Of the 123 samples submitted to the diagnostic laboratory over four months in 2024, 15 tested positive via RT-PCR. All 15 were detected by both RADs, indicating a diagnostic sensitivity of 100%. Initially, 9 of the 108 RT-PCR-negative samples tested weakly positive on the CerTest but only 5 of these were confirmed to be positive on repeat testing. One of the five samples also tested positive on the FastTest. Overall, compared to the RT-PCR, the CerTest and the FASTest had a specificity of 95.6% and 99.1%, respectively. This resulted in a concordance rate of 95.9% for the CerTest and 99.2% for the FastTest. These results are summarised in Table 4 and Table 5 and sample information and results are presented in Supplementary Table S2.

## 4. Discussion

This paper describes the use of 263 field samples (140 freeze-thawed and 123 fresh samples) to compare two techniques commonly used for the diagnosis of equine rotavirus. A variety of diagnostic techniques have been used to detect rotavirus in the faeces of foals, including electron microscopy (EM), virus isolation in the presence of proteolytic enzymes such as trypsin, enzyme-linked immunoassays (ELISAs), rapid antigen detection (RAD) tests based on immunochromatography or latex agglutination, and RT-PCR [19,20,24,25]. Early detection is essential to instigate the appropriate treatment of affected foals and their isolation from other foals. Thus, the more traditional techniques such as EM and VI have been superseded by RADs and RT-PCR as the frontline diagnostic tests for equine rotavirus diarrhoea [24,26,27]. This study compared the diagnostic performance of two RADs, the FASTest and the CerTest, with that of an in-house real-time RT-PCR. The FASTest is marketed for the detection of rotavirus A antigens in the faeces of animals but the CerTest is recommended only for the testing of human faeces. However, such human test kits are frequently used for testing equine patients [19].

The sensitivity of RADs in comparison to RT-PCR is sometimes questioned as some RADs for other viral infections such SARS-CoV-2 have proven to be several thousand times less sensitive than PCR [28]. In this study, testing of serial dilutions of clinical faecal samples confirmed the superior sensitivity of real-time RT-PCR which is considered the gold standard test for the detection of rotavirus. The higher limit of detection for RT-PCR when compared to RADs is of clinical significance for veterinary practitioners and scientists in that the RADs may not detect animals shedding low quantities of the virus. Thus, samples from suspect cases that test negative on a RAD should be referred for real-time RT-PCR testing in a laboratory. An evaluation of RAD kits for the diagnosis of equine rotavirus in Japan using 249 faecal samples demonstrated that the kit with the highest sensitivity compared to RT-PCR was 81.9% and its specificity was 98.2%, resulting in a concordance rate of 92.8% [24]. In this evaluation of two RADs commercially available in Europe and not included in the Japanese study [24], the results of 722 RAD tests and 361 RT-PCR tests were analysed, and the diagnostic performance of both kits also had a high level of concordance with RT-PCR (>95%). The FASTest had a higher level of concordance with RT-PCR than the CerTest and it was also more sensitive than the CerTest Rotavirus kit on testing serial dilutions of faeces samples.

Real-time RT-PCR is laboratory based and requires specialised equipment and reagents. RT-PCR is suitable for multiple-pathogen testing that is necessary for differential diagnosis, and for high-throughput screening. The latter is extremely useful during an outbreak for screening a population, assessing the level of challenge and monitoring viral spread. Given its sensitivity, real-time RT-PCR is also the test of choice for samples of possible low viral-abundance, for example, screening clinically recovered foals prior to movement when the diarrhoea has resolved but virus shedding may persist for several days. In such situations, it is important to use the most sensitive test available and to take all necessary precautions to mitigate disease spread to other premises. Similarly, RT-PCR is suitable for testing foals with severe watery diarrhoea, and older mildly affected foals that may have a lower viral load in their faeces [20].

In contrast to real-time RT-PCR, the RADs for rotavirus can be performed on the farm as they do not require laboratory facilities with specialist equipment and trained personnel. They are focused on the diagnosis of individual cases and if reliable, are suited for preliminary assessment and determination of the urgency of the appropriate treatment and isolation. RADs such as FASTest and CerTest are potentially useful for the rapid screening of foals prior to entering a veterinary hospital, farm or quarantine facility where the introduction of an infected animal could precipitate an outbreak. Also, as the primary therapy for rotavirus-induced diarrhoea is fluid replacement, a rapid diagnosis may contribute to minimising the unnecessary use of antibiotics and the development of antimicrobial resistance.

It is widely recognised that point-of-care (PoC) tests such as RADs have the potential to significantly augment laboratory-based diagnostic assays enabling the early detection and containment of economically significant animal diseases. A BioSpace report published in 2022 estimated that the Veterinary PoC test market will grow at a 12.3% rate from 2021 to 2030 to be worth $5.69 billion by 2030 [22,29]. The acceptance of the role of PoC technology in responding to emerging diseases has been demonstrated by the WOAH (World Organisation for Animal Health, formerly OIE) Network for African Swine Fever (ASF) in an overview of ASF diagnostic tests for field application [30]. However, PoC tests are often not validated or regulated to the same standard as laboratory-based assays [22,31], for example, some commercially available PoC tests for rabies have been shown to be inadequately sensitive and not fit for purpose [32]. All commercial kits included in the WOAH Register are certified as validated and fit for purpose but there are currently few PoC tests included in the Register. Given the current limitations of RAD test validation and the lack of regulatory oversight in many countries [22], diagnostic evaluations such as those presented here are essential for identifying reliable PoC tests and promoting confidence in their use as screening tests. This study provides some reassurance that the two RADs tested may be valuable portable tools to detect equine rotavirus in foals before laboratory confirmation. Such tests are an adjunct to, not a replacement for laboratory testing. Negative samples from suspect cases and weak positives should always be submitted to a specialist laboratory for real-time RT-PCR testing.

## Figures and Tables

**Table 1:** Detection limits of RADs and real-time RT-PCRs in serial ten-fold dilutions of clinical samples.

	Sample Dilutions					
Test	Sample	Neat	10^−1^	10^−2^	10^−3^	10^−4^
CerTest	A	**+++**	**+++**	**+**	**-**	**-**
	B	**+++**	**++**	**-**	**-**	**-**
	C	**+++**	**-**	**-**	**-**	**-**
	D	**+++**	**-**	**-**	**-**	**-**
FASTest ROTA	A	**+++**	**+++**	**+++**	**-**	**-**
	B	**+++**	**+++**	**++**	**-**	**-**
	C	**+++**	**++**	**-**	**-**	**-**
	D	**+++**	**++**	**-**	**-**	**-**
Real-time RT-PCR	A	**+++**	**+++**	**+++**	**+++**	**-**
	B	**+++**	**+++**	**-**	**-**	**-**
	C	**+++**	**+++**	**+++**	**+**	**-**
	D	**+++**	**+++**	**+++**	**+++**	**-**

+, ++ and +++ denote equine rotavirus A positives on one, two and three tests, respectively. - indicates equine rotavirus A-negative results for all three tests.

**Table 2:** Detection of equine rotavirus A in freeze-thawed faecal samples by CerTest compared with real-time RT-PCR.

CerTest Results	Real-Time RT-PCR		Total	Sensitivity	Specificity	Concordance Rate
	+	-				
+	64	3	67	95.7%	96.1%	95.7%
-	3	70	73			
Total	67	73	140			

+ denotes equine rotavirus A positive, - indicates equine rotavirus A negative.

**Table 3:** Detection of equine rotavirus A in freeze-thawed faecal samples by FASTest compared with real-time RT-PCR.

FASTest ROTA Results	Real-Time RT-PCR		Total	Sensitivity	Specificity	Concordance Rate
	+	-				
+	62	0	62	93.1%	100%	96.4%
-	5	73	78			
Total	67	73	140			

+ denotes equine rotavirus A positive, - indicates equine rotavirus A negative.

**Table 4:** Detection of equine rotavirus A in fresh faecal samples by CerTest compared with real- time RT-PCR.

CerTest Results	Real-Time RT-PCR		Total	Sensitivity	Specificity	Concordance Rate
	+	-				
+	15	5	20	100%	95.6%	95.9%
-	0	103	103			
Total	15	108	123			

+ denotes equine rotavirus A positive, - indicates equine rotavirus A negative.

**Table 5:** Detection of equine rotavirus A on fresh faecal samples by FASTest compared with real-time RT-PCR.

FASTest ROTA Results	Real-Time RT-PCR		Total	Sensitivity	Specificity	Concordance Rate
	+	-				
+	15	1	16	100%	99.1%	99.2%
-	0	107	107			
Total	15	108	123			

+ denotes equine rotavirus A positive, - indicates equine rotavirus A negative.

## Data Availability

The original contributions presented in this study are included in the article/Supplementary Material. Further inquiries can be directed to the corresponding author(s).

## Supplementary Materials

The following supporting information can be downloaded at: https://www.mdpi.com/article/10.3390/v17030413/s1, Table S1: Details of 140 archived faeces samples tested by RT-PCR and RAD kits; Table S2: Details of fresh faecal samples tested by RT-PCR and RAD kits. Figure S1a–h: Photographs of preliminary RAD results included in the sensitivity investigation.
