# Supplementary material for: Diagnostic Performance of Rapid Antigen Tests to Detect Equine Rotavirus A
Source: Viruses. 2025 Mar 14;17(3):413. doi: 10.3390/v17030413 (PMC11946396; doi:10.3390/v17030413)
Supplement: Supplementary file 1 [file viruses-17-00413-s001.zip › Supplementary Figure S1a-h.pdf]

Supplementary Figure S1

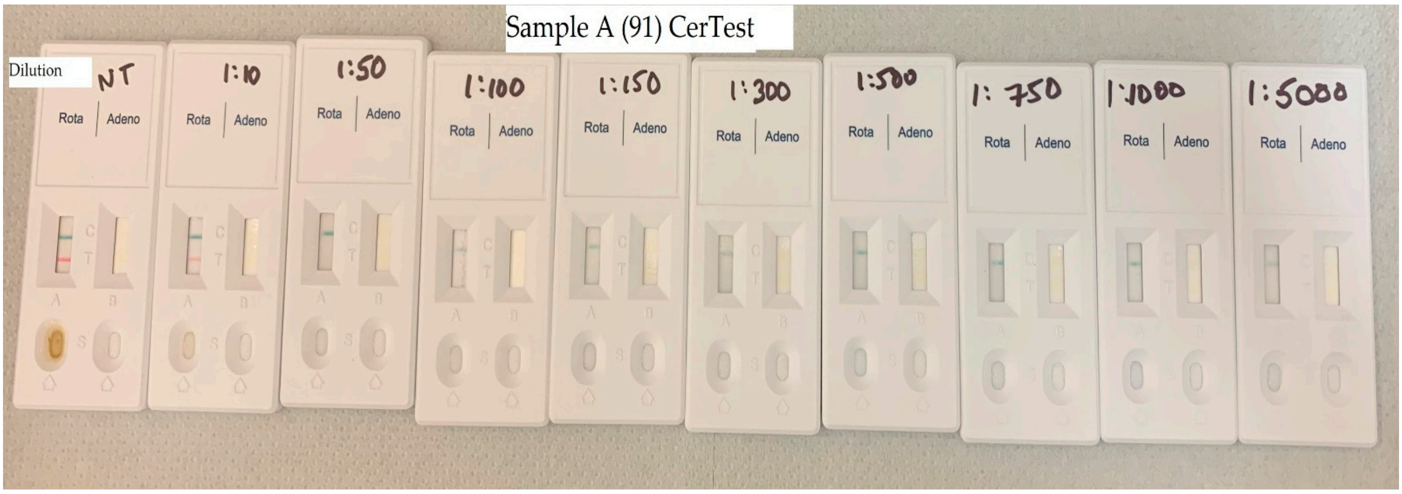

Figure S1a

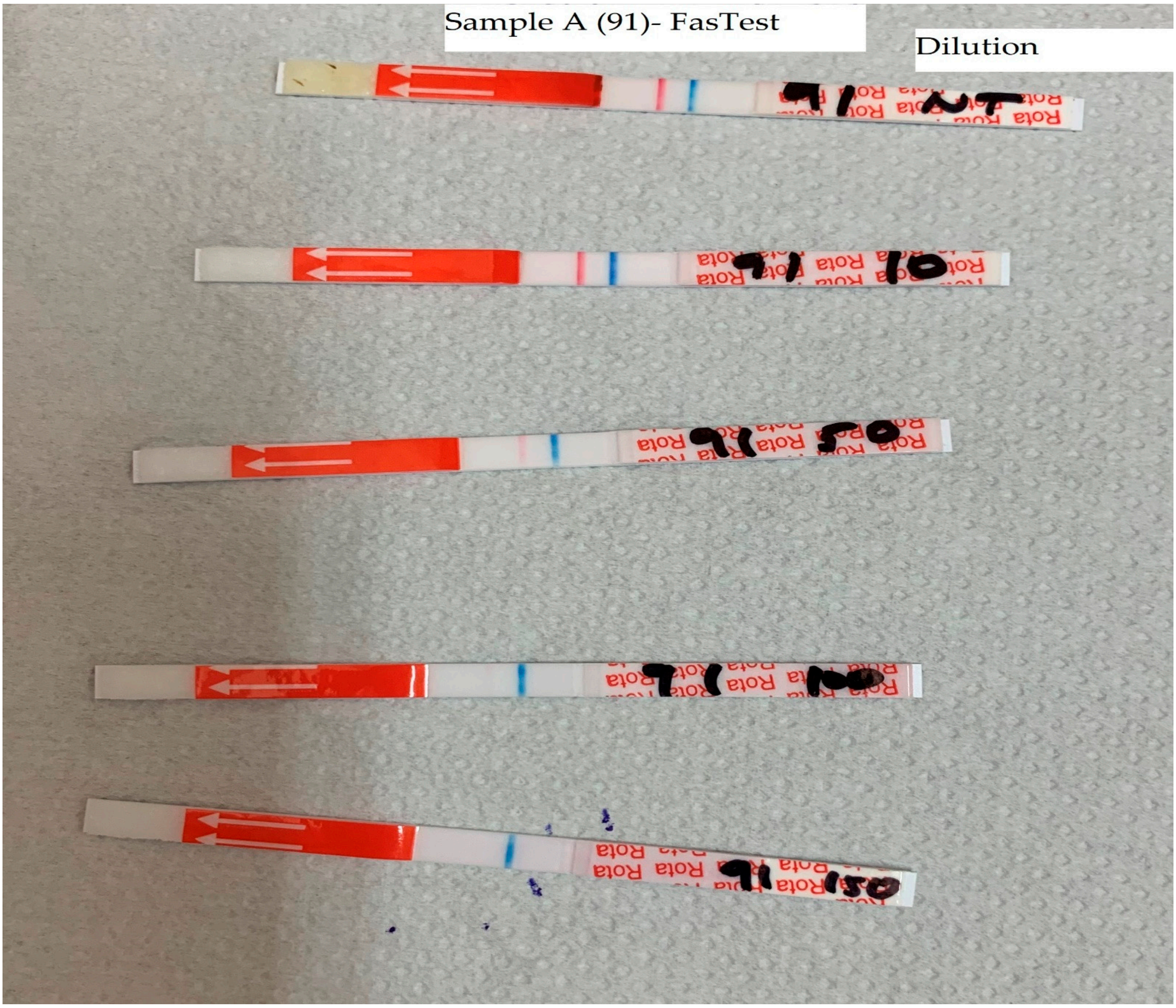

Figure S1b

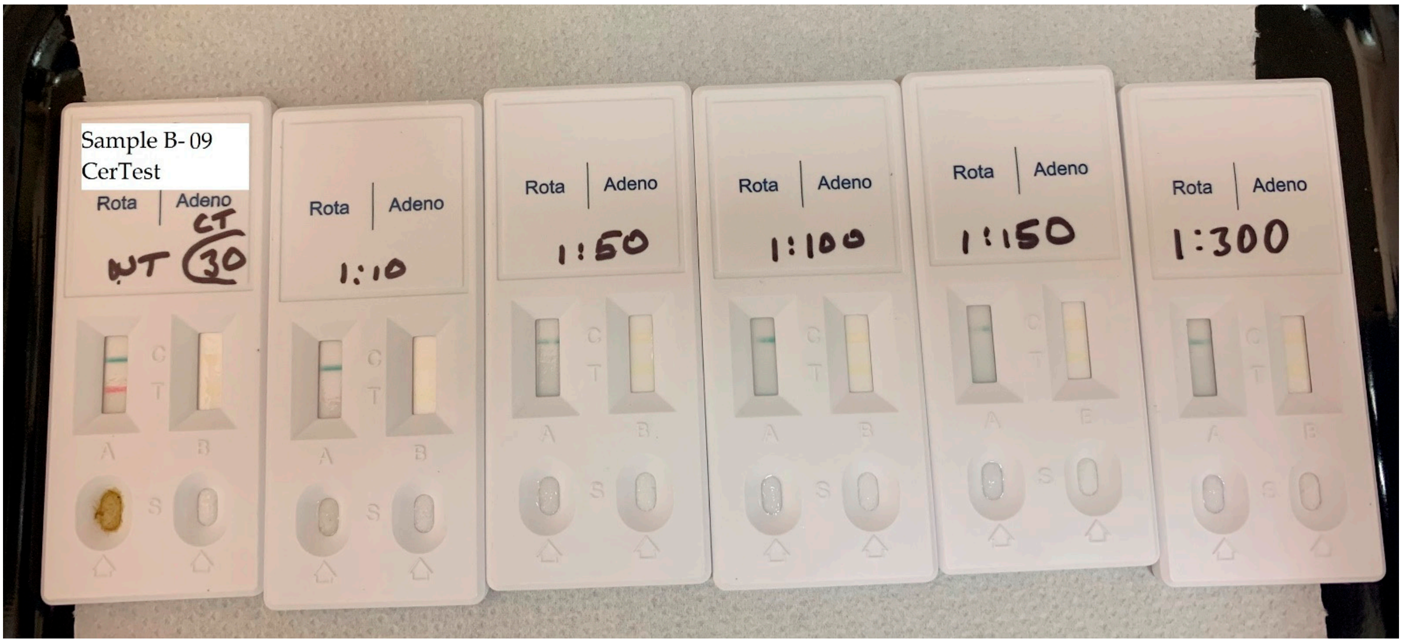

Figure S1c

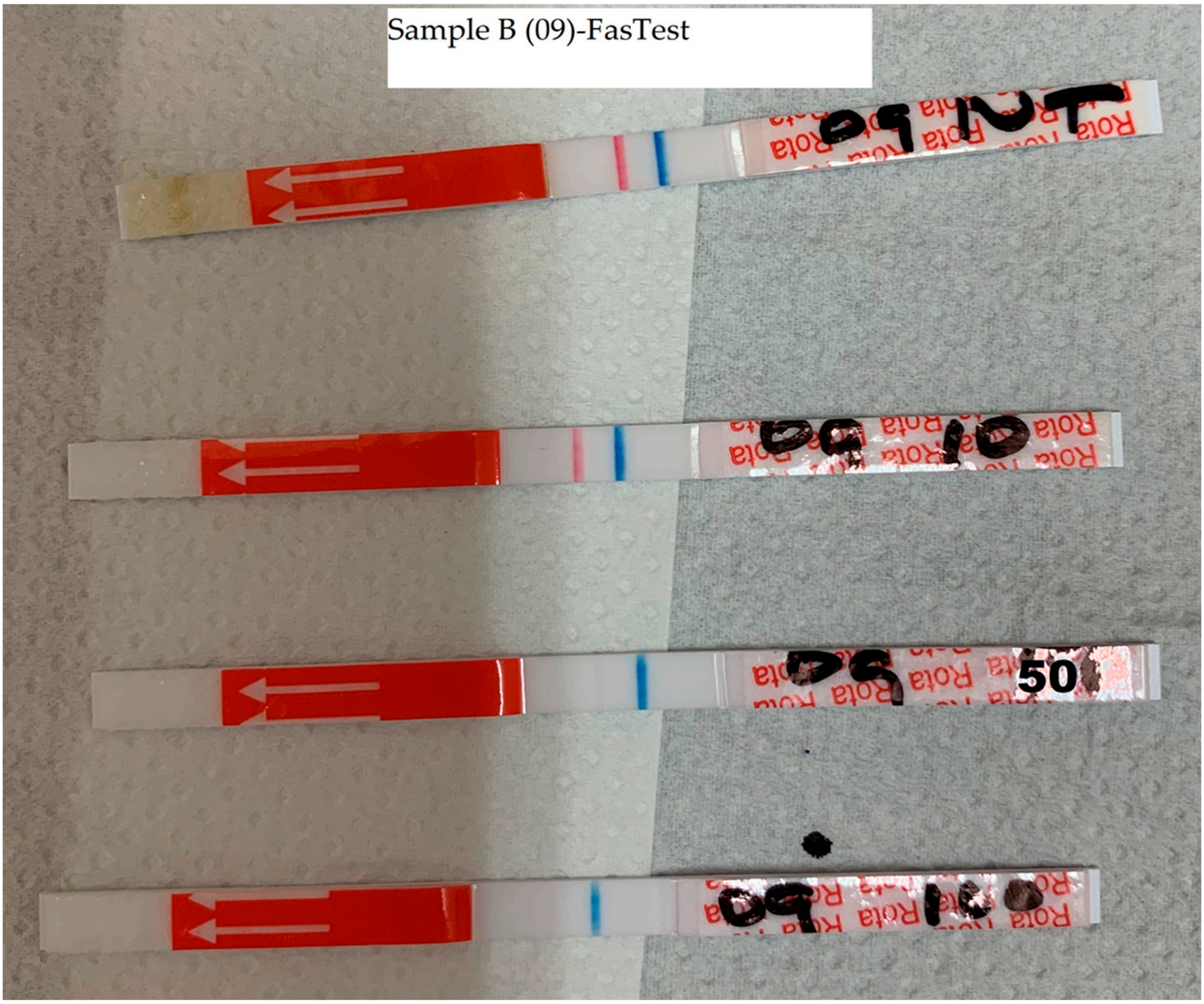

Figure S1d

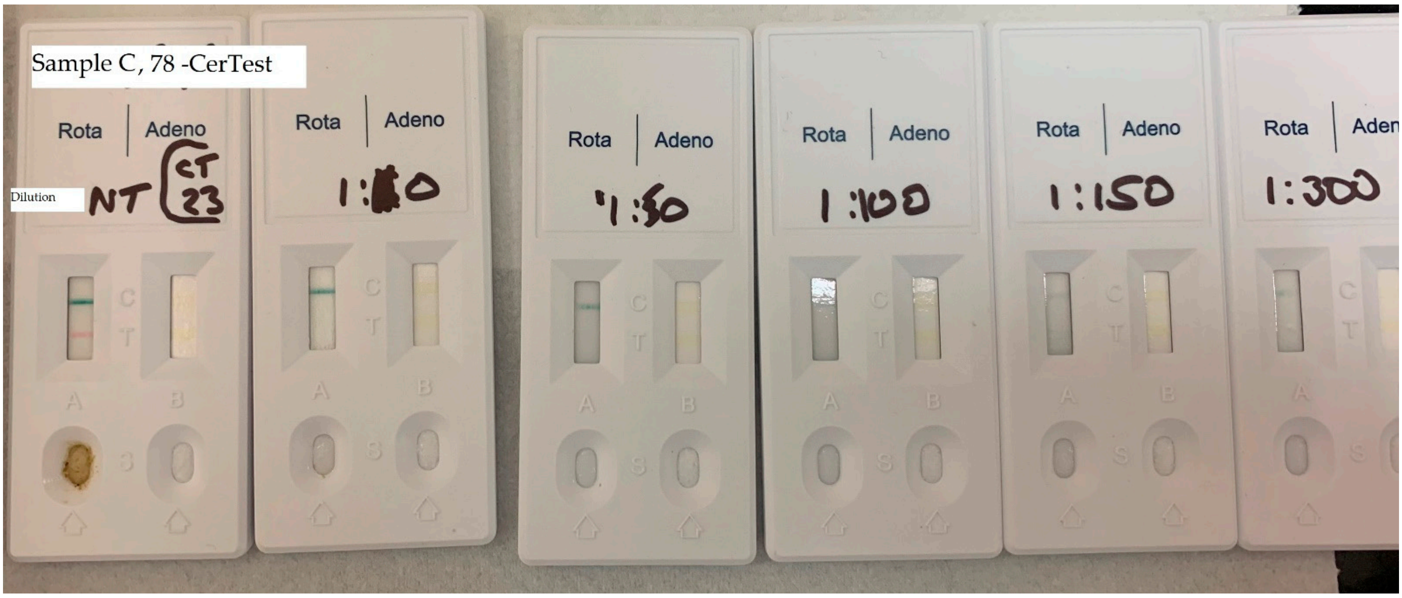

Figure S1e

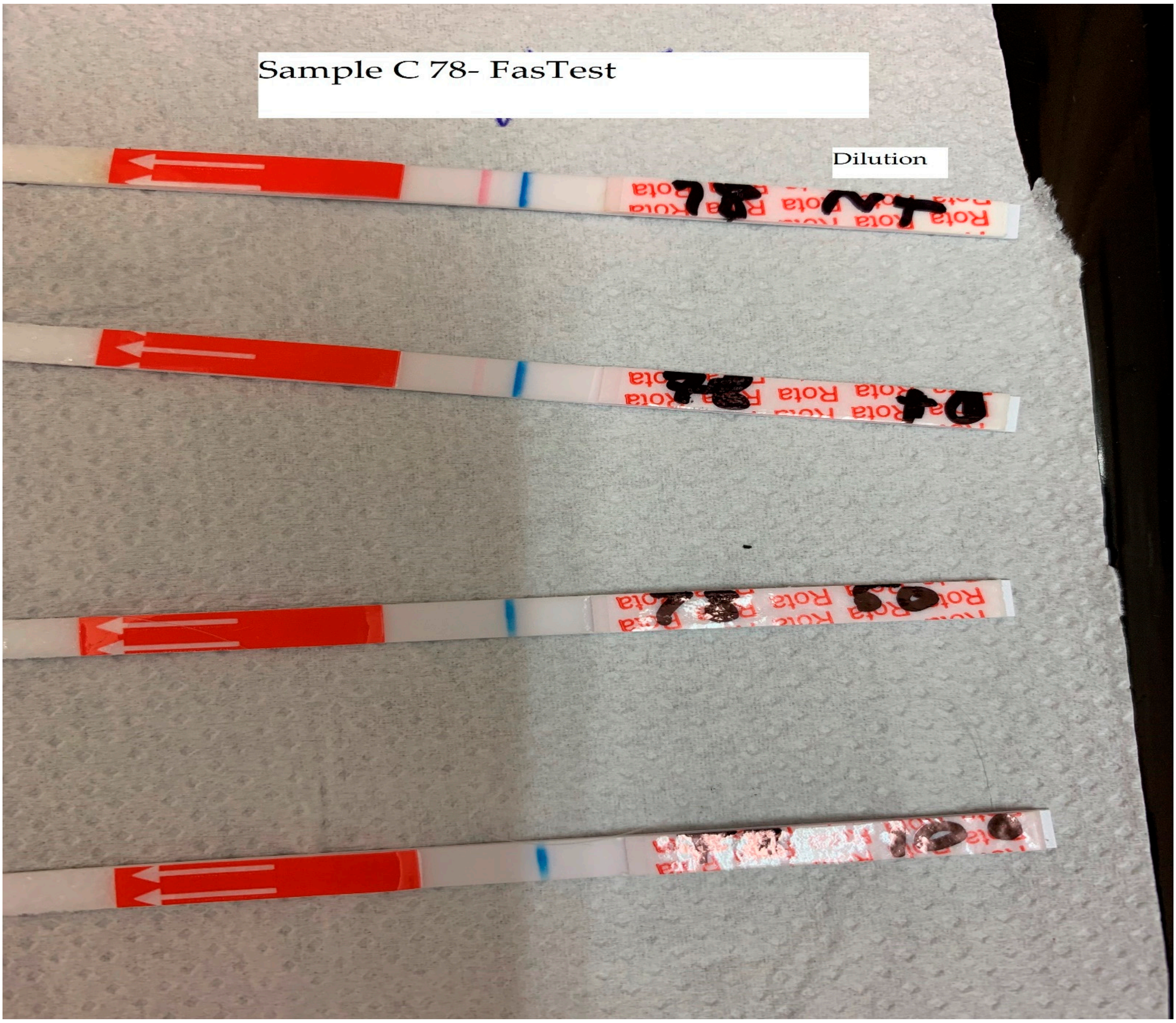

Figure S1f

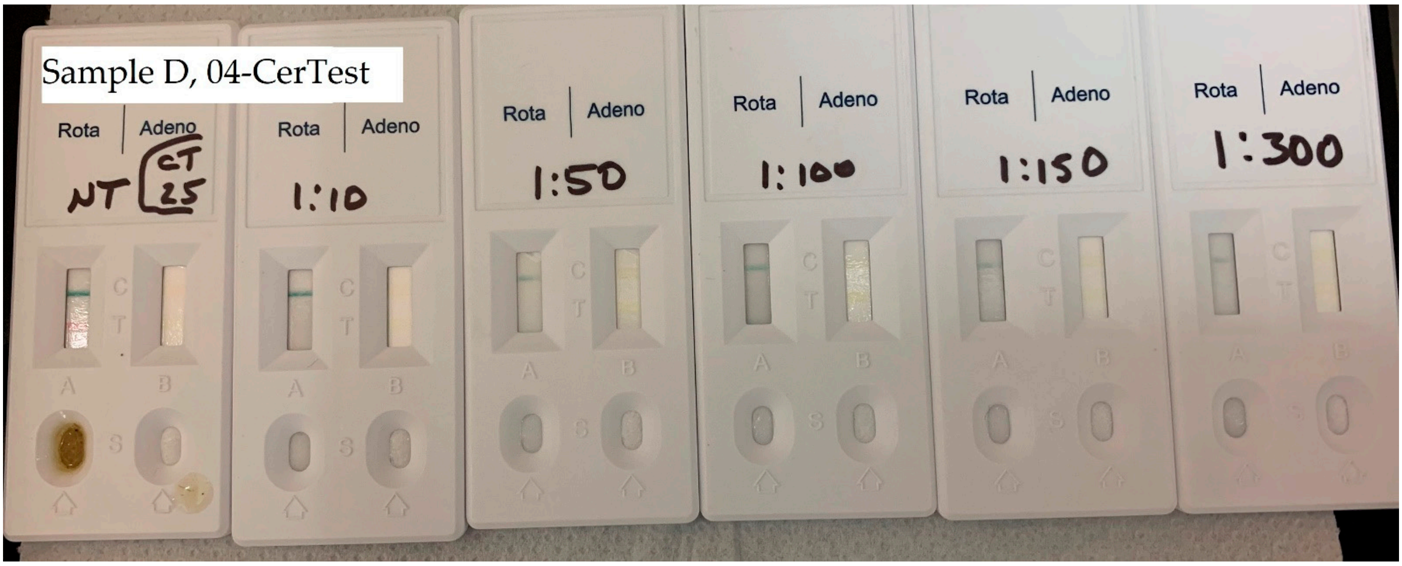

Figure S1g

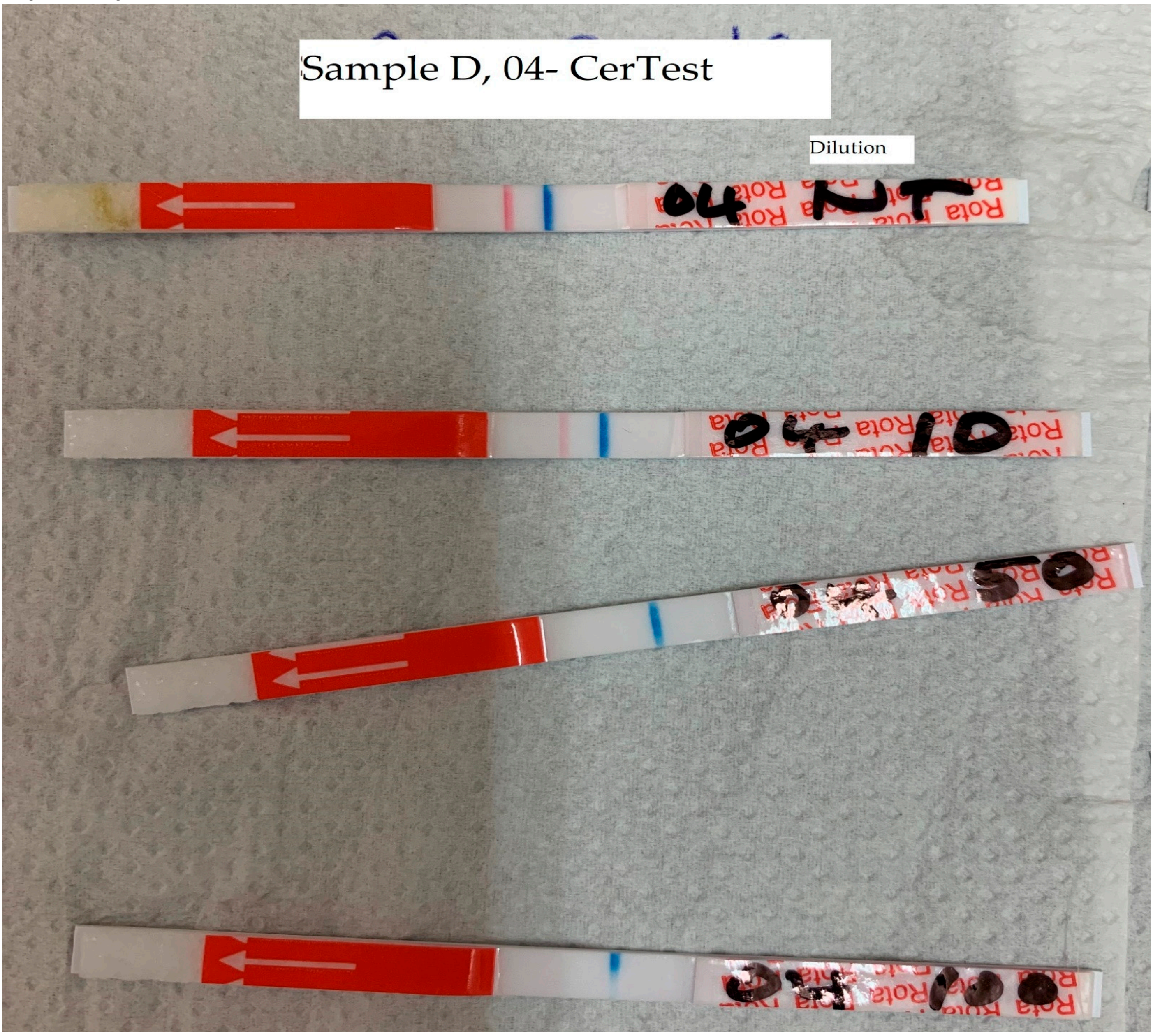

Figure S1h
